# Food Security Review Based on Bibliometrics from 1991 to 2021

**DOI:** 10.3390/foods11233915

**Published:** 2022-12-04

**Authors:** Junfang Li, Wei Song

**Affiliations:** 1 Key Laboratory of Land Surface Pattern and Simulation, Institute of Geographic Sciences and Natural Resources Research, Chinese Academy of Sciences, Beijing 100101, China; 2 School of Earth Science and Resources, Chang’an University, Xi’an 710054, China; 3 Hebei Collaborative Innovation Center for Urban-Rural Integration Development, Shijiazhuang 050061, China

**Keywords:** food security, bibliometrics, sustainable development, thematic analysis

## Abstract

Food security is related to human wellbeing and sustainable development and an important guarantee for world peace. In the context of global climate change, increased food demand, resource depletion, conflicts, and frequent public health emergencies, food security is widely seen as one of the top challenges. Food security research has obvious interdisciplinary characteristics, involving a wide range of fields. We analyzed the literature on food security in the Web of Science core collection from 1991 to 2021, using bibliometric methods with the aid of the Biblioshiny software package. By collecting, screening, analyzing, and visually expressing the literature data, the following conclusions were drawn: (1) In the past 30 years, the annual number of publications on food security increased. The period can be divided into three stages: 1991–2003 as the budding period, 2004–2012 as the development period, and 2013–2020 as the high-yield and active period. The top three journals discussing food security issues are *Food Security*, *Sustainability*, and *Food Policy*, and these journals focus on the publication of comprehensive views from interdisciplinary perspectives. (2) Studies on food security cover 138 countries or regions. The top three countries in terms of the number of published articles are the United States, the United Kingdom, and China. Among the top 20 countries in terms of the number of published articles, European countries are highlighted. (3) Climate change, food security, agriculture, policy, and management are the other high-frequency keywords in the field of food security; climate change occurred 321 times. The word sub-Saharan Africa also occurred more frequently, indicating that food security in sub-Saharan Africa has attracted wide attention. (4) The food security theme mapping clearly showed the research status and future development trends of various topics in the field. Currently, food production, climate change, and sustainable development are the most popular themes. Research on food sovereignty, ecological agriculture, child obesity, and other aspects is an emerging field. (5) We predict that in the future, the field of food security may focus on the expansion and improvement of the food security evaluation system, the balance between sustainable development and food security goals, the improvement of agricultural production and management efficiency, and the research on government policies and strategies. Our results provide a reference for grasping the current situation, key research direction, and development trend in the field of food security.

## 1. Introduction

Food security concerns the stability of the international community and human wellbeing and plays an important role in sustainable development. The 2030 Agenda for Sustainable Development lists hunger eradication, achieving food security, and improving nutritional status as high-priority areas in the 17 Sustainable Development Goals [1]. In recent years, with the frequent occurrence of wars, extreme weather events, and public security incidents, global food security issues have become the focus of attention [2,3]. The innovation and development of science and technology promoted grain production in countries around the world, but the huge population base still brings great challenges to the carrying capacity of food resources. The number of people globally affected by hunger increased from 768 million in 2020 to 828 million in 2021 [4], highlighting the worrying situation of global food security.

Currently, research on food security mainly includes the connotation of food security, the influencing factors, and how to guarantee food security. In 1974, the UN Food and Agriculture Organization first defined food security as “we should ensure that anyone anywhere can get enough food for survival and health in the future” [5] (pp. 303–320). The definition of food security, as agreed upon at the 1996 World Food Summit, contains four aspects: food, access, use, and stability. With the deepening of research, the concept of food security has been continuously refined and expanded, taking food hygiene, politics, human rights, and social culture into consideration. Therefore, food security not only refers to the problem of adequate food supply but also involves a balanced regional distribution and nutrition as well as stable eating habits. In 2012, the FAO updated the definition of food security, completing the development from only meeting survival needs to meeting positive and healthy living and food preferences [6]. The concept of food security from the initial emphasis on “food supply” to the later “focus on family and individual food acquisition power” indicates the extension from the macro overall to the micro individuals. After the 2012 revision, the concept of food security included macro- and micro-aspects, quantity and quality, and supply and demand. It is predicted that by 2050, food production needs to increase by 70% to meet the demands of the growing population. Currently, about 750 million people in the world face food security problems, and 1 billion people face long-term malnutrition [7].

Therefore, based on the macro-background of global climate change, wars and social conflicts, the uneven spatial distribution of water and soil resources, and the declining land production potential, several explorations from different perspectives and levels have been performed. Climate change is one of the important triggers of global food security, with complex impact mechanisms. The extent to which food insecurity can be attributed to climate change was quantitatively assessed by Dasgupta and Robinson [8]. Climate change will not only affect the global food supply directly by affecting food production but will also further push up food prices, which will exacerbate food security inequalities. Many efforts have been made at the national level to ensure the food security of certain countries. For example, in the face of food security threats caused by the decline in the rural population [9], rural aging [10,11], and abandoned farmland [12,13], China has actively adopted relevant policies and measures, such as strictly observing the red line of cultivated land (referring to the minimum land area for regular cultivation; China’s current red line is 120 million hectares of cultivated land), the construction of high-standard farmland, and the comprehensive improvement of rural land [13,14]. Policies and the international environment have a significant impact on regional food security. Recently, the emergence of the COVID-19 pandemic, conflicts between Russia and Ukraine, and other crises have had a huge impact on international grain trade and sounded the alarm for some countries and regions that rely heavily on grain imports. For example, Singapore’s food supply chain was seriously threatened [15] by these incidents. Although food security in Asia and Latin America has improved due to the development of science and technology, improved agricultural productivity, and government intervention, the problem of regional distribution inequality remains. At present, the food risk in some developing countries in Africa, South Asia, and other regions is still high [16].

On the research scale, food security has global, national, family, and individual aspects. At the national level, food security is deeply affected by the population base, the population growth rate, and the urbanization level. Countries at different stages of development have different priorities for ensuring food security. For example, developed countries focus on the issue of regional food security balance, whereas developing countries, such as Africa, are committed to eliminating the food threat posed by poverty [17]. National food security is an important guarantee for food security at the family level. Low family income, large gaps between rich and poor, and immature markets will all lead to family food insecurity. Personal food security is highly important for personal nutrition and health, and excessive nutrient intake can cause many diseases, such as childhood obesity [18]. Analyzing the factors affecting food security is necessary to find adequate solutions. The influencing factors have been widely studied, and an evaluation index system has been established.

Research in the field of food security involves multiple disciplines and the comprehensive analysis of food security-related research from an interdisciplinary perspective to obtain an in-depth understanding of the current research situation. After Pritchard proposed the term bibliometrics in 1969, bibliometrics attracted global attention, which was most evident in the late 1970s. Bibliometric research mainly focuses on three fields: methodology, scientific information, and scientific policy, of which the first is the basic research field [19]. With the gradual maturity of bibliometric methods, this approach has been widely used in the quantitative analysis of literature information in various disciplines. In the process of practical application, it has been continuously expanded and extended, promoting the generation and development of scientific measurement methods, information measurement methods, and network information measurement methods. Because of the great significance of clarifying the development context of food security and putting forward the dual goals of food security and sustainable development, we conducted a quantitative analysis of the number of articles, authors, institutions, and keywords in the field of food security. The research questions were as follows:

(1) Which topics containing food are the most popular in academia?
(2) What is the current state of global cooperation on food security?
(3) How do the keywords in food security research cluster together?
(4) What areas of food security need further research?

## 2. Data Sources and Research Methods

### 2.1. Data Sources

Web of Science is the largest and most comprehensive collection of information resources in the world and contains more than 12,000 authoritative and high-impact academic journals in the fields of natural sciences, engineering, and biomedicine. We used the Web of Science core collection from the Web of Science database as the data source; the search method was the Topic Subject (TS) search, and the language was English. The search formula is TS = (“Food Security” or “Grain Security”). After deduplication and screening, 3734 documents in the field of food security, published between 1991 and 2021, were obtained, including 2832 research papers; 64 conference papers; 402 review articles; and 436 books, book chapters, letters, and other types of documents.

### 2.2. Research Methods

#### 2.2.1. Methods of Bibliometric Analysis

Bibliometry, a traditional quantitative analysis method in the field of library and intelligence science, originated during World War II. After a long development period, it has been used in many disciplines and has become an important analysis method in scientific research. We combined bibliometric and other measurement research methods to process the retrieved literature information. The resulting data were further explored and analyzed using the software packages Bibliometric and VOS viewer and finally visualized for expression. This paper mainly observed and interpreted the overall literature data in the field of food security based on the publication volume evolution trend analysis, historical citation analysis, popular journal analysis, high-yield author analysis, main research country/institution analysis, keyword analysis, and theme analysis.

#### 2.2.2. Research Ideas

**Figure 1:**
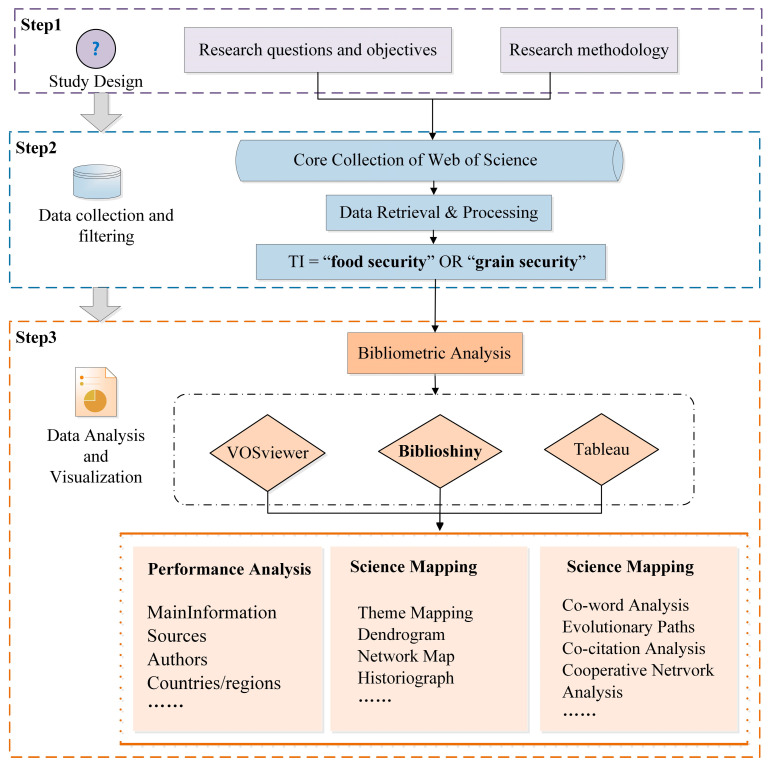
Measurement and analysis design process in the field of food security.

The first step in a research review is conducting a research design, including raising research questions, clarifying research objectives, and identifying research methods (Figure 1). The second step is data collection and screening, using the literature research method for data collection, screening, and processing after collection. The third step is data analysis and visual expression. We first imported the data into Bibliometric for analysis, analyzed the input of food security research subjects (authors, journals, institutions, among others), and then discussed and analyzed the research hot-spots and preface frontiers combined with VOS viewer.

## 3. Analysis of Results

### 3.1. Evolution Trend of Publication Volume

**Figure 2:**
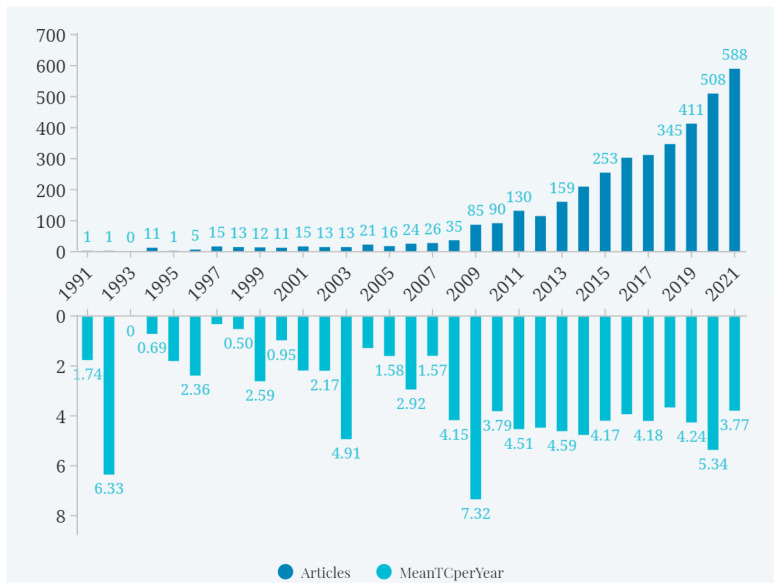
Number of published and cited food security research articles from 1991 to 2021.

The number of documents is an important indicator of the development stage of a certain field and a reference standard to predict the future trend. Figure 2 shows the development trend of research in the field of food security based on an analysis of the distribution of annual publications. From 1991 to 2021, the overall number of food security documents increased, with an average annual growth rate of 23.68%. Food security research can be roughly divided into three stages: In the first stage (1991–2003), the number of publications was small and the growth was slow. The field of food security had not yet received wide attention from the academic community, and the related studies were mainly focused on qualitative analysis. However, some pioneering articles laid the foundation for food security research. For example, an article published in 1992 obtained 190 citations, with an average annual reference volume of 6.33 [20]. In the second stage (2004–2012), more scholars began to focus on food security involving cultural, political, social, and other aspects, and this field received considerable interdisciplinary attention. Scientists began to discuss food security issues from different angles, such as transgenic technology, bringing opportunities and challenges to food security. In the third stage (2013–2021), the number of articles increased rapidly, making this stage one of high yield and activity. Food security attracted wide attention and gradually became an important issue.

### 3.2. Citation Analysis of Food Security Studies

By analyzing the local and global citation rates of the articles in the field of food security, we found that most of the top-cited articles were published around 2010, indicating that the research results in the field of food security during this period were more influential (Table 1). The article with the most local citations (LCs) was one by Professor Per Pinstrup-Andersen of Cornell University, published in *Food Security* in 2009, on the issues of “how to define food security” and “how to measure food security” [21]. The second-ranked article in terms of LCs synthesized the perspectives of the social and natural sciences; constructed a theoretical framework for the interaction of food security, ecosystem services, and social welfare; and revealed the key processes and main factors that controlled food through the elaboration of integrated systems of food production, supply, access, and use [22]. The establishment of this theoretical framework laid a foundation for future studies of the impact of global environmental changes on food security. The numbers of local citations of the above two articles both exceeded 100, indicating outstanding contributions in the field of food security. The third-ranked article in terms of LCs analyzed the definitions, conceptual frameworks, and household food security indicators of food security, defining food security as a global, national, family, and personal multifaceted concept. To better monitor and measure household food security, the advantages and disadvantages of different indicators at the specific level of analysis were evaluated [23]. Dana [24] called for global attention to be paid to phosphorus, an important raw material needed for food production, and suggested the inclusion of long-term phosphorus deficiency in the global food security priority agenda. The article obtained 2722 global citations, indicating that this research was widely recognized. Most of the top 10 articles in local citations were studies on the definition of food security [21], the conceptual framework [22], and the measurement index [23,25]. In addition, publications on food security impact factors, climate change and food security, and the impacts of food losses and waste on resources such as chemical fertilizers and fields [26,27] also attracted more attention.

**Figure 3:**
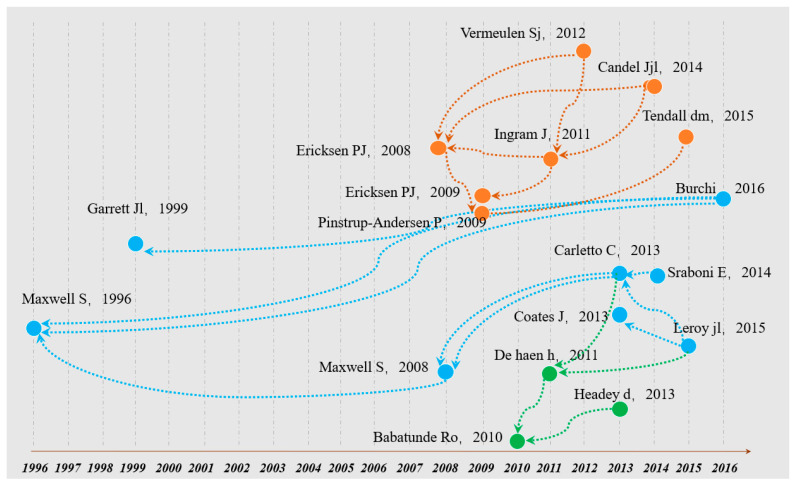
Historical direct citation network of papers published from 1996 to 2021 in the field of food security.

In Biblioshiny, 20 nodes were selected, representing seminal work within the domain and some classical studies (Figure 3). The earliest node in the literature on food security was an article published in 1996 in *Food Policy*, entitled “Food security: a post-modern perspective” [28], with three different reference chains, ranking fourth with 56 LCs. This article identified three major shifts in food security thinking from the world and the country to families and individuals, from food first to livelihood, and from objective indicators to subjective perception. An interdisciplinary article by Dana [24], published in *Global Environmental Change*, revealed the possibility of a phosphate shortage in the future, emphasizing the importance of scientifically managing the phosphorus resources in the global food system.

### 3.3. Popular Journal Analysis

Academic journals all have their own purpose, scope, focus, and research interests. Popular journals not only represent the recognition and choice of most researchers in this field but also are an important way to obtain hot research content. Researchers can also accurately locate their research results by interpreting articles published in popular journals. According to Bradford’s Law, the concentration and dispersion of papers in the food security field from 1991 to 2021 were analyzed; the periodicals were arranged in descending order according to the number of papers published in the field of food security and divided into core, related, and unrelated areas. The numbers of journals in the core areas, related areas, and unrelated areas have the relationship 1: n: n^2^. The core area was composed of seven journals; the journal with the largest number of articles was *Food Security*, and that with the highest H-index was *Food Policy* (Table 2).

**Figure 4:**
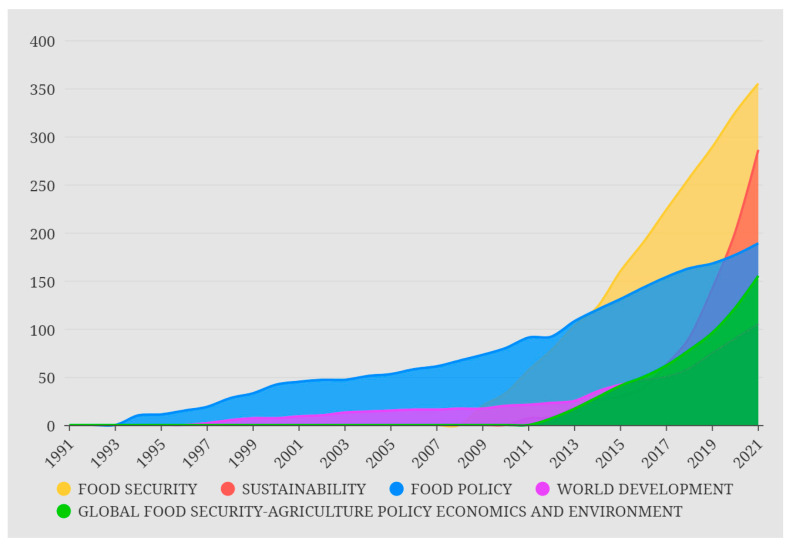
Popular journals in the field of food security from 1991 to 2021.

*Food Security* is an interdisciplinary international journal focused on exploring global food security issues. The journal pays special attention to the publication of a comprehensive view of food production, agricultural development, access to food and nutrition science, sociology, and economics. *Food Security* and *Sustainability* have seen a high growth rate of publications in recent years, and their cumulative publications surpassed those of *Food Policy* in 2014 and 2019, respectively, making them the most popular journals in the field (Figure 4).

### 3.4. Analysis of the Distribution Characteristics of the Major Research Countries and Institutions

**Figure 5:**
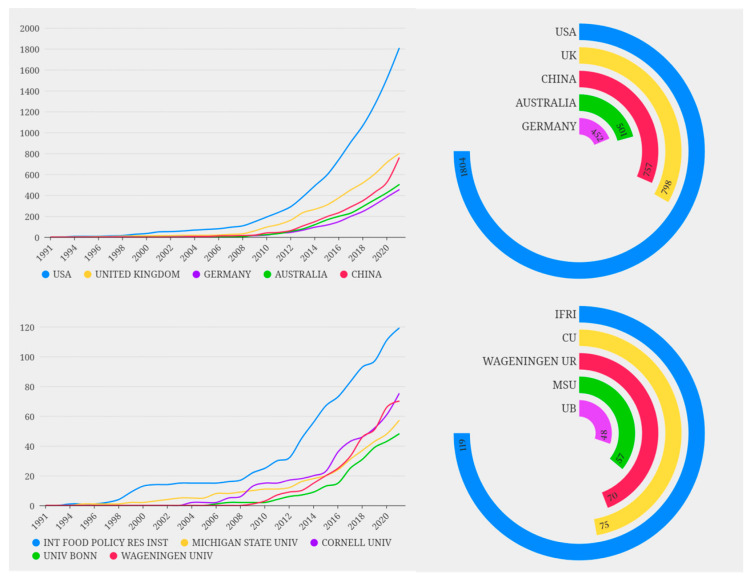
Top 10 countries and institutions publishing in the field of food security. Note: IFPRI: International Food Policy Research Institute; CU: Cornell University; WA-GENINGEN UR: Wageningen University & Research; MSU: Michigan State University; UB: Rheinische Friedrich-Wilhelms-Universität Bonn.

The dataset used for the analysis of food security studies contained publications from 134 countries/regions (Figure 5). European countries accounted for the majority of the top 20 countries, followed by countries in the Americas. The United States had most of the publications, with 1804 articles, about twice the number of the UK, which had the second highest number of publications. The number of US publications also increased more rapidly than that of the other countries, suggesting that the US was at the forefront of food security research. China ranked third in the number of articles, with 757 articles, with a high growth rate from 2020 to 2021. Because of the basic conditions of China, with a higher population and a relatively low land area, food security received high attention, with many publications in the fields of ensuring the ability to obtain food [29], forecasting the demand for food and nutrition security [30], and balancing the supply and demand structure of food [31].

The agency with the greatest publication output is the International Food Policy Research Institute (IFPRI), with 119 articles. It is an international organization dedicated to providing research on food security, hunger eradication, and poverty and is a benchmark research institute in the field of food security. It is followed by Cornell University and Wageningen University, with 75 and 70 papers, respectively (Figure 5). The former and the latter are the world’s top agricultural and environmental science research institutions and enjoy a high international reputation. The University of Michigan ranked fourth in publications and was responsible for some high-quality articles that were highly influential in the field. For example, an article on the assessment of food security indicators, published in *Advances in Nutrition* in 2013, received 339 citations [32]. The agencies that published the largest numbers of papers were mostly located in the United States, the Netherlands, and Germany.

### 3.5. Keyword Analysis

**Figure 6:**
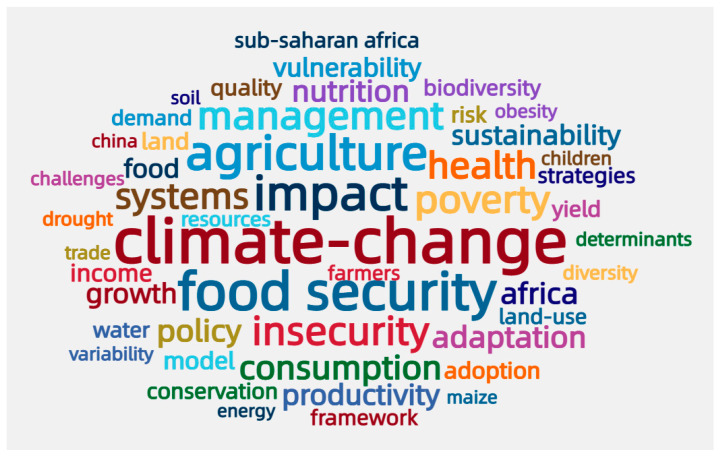
High-frequency keywords in the field of food security (top 50 keywords).

Keywords are a high-level summary of the topic of the article, and analyzing keywords in the field can quickly and accurately capture research hot-spots. Regarding the field of food security, the frequency of individual keywords represented the number of research findings. Climate change was the most frequently encountered keyword and was detected 321 times. Climate change directly affected the four pillars of food security (quantity, availability, usability, and stability) and had the most extensive impact on food security. The second most frequently occurring keywords were agriculture, policy, management, poverty, and insecurity, indicating that these aspects were highly related to food security. Sub-Saharan Africa (68 times), China (50 times), and India (27 times) appeared as keywords, showing that these countries were the focus of food security research (Figure 6). For a long time, the issue of food security in sub-Saharan Africa has received extensive attention from the international community. Substantial research was undertaken to identify existing problems in food security [33], eliminate regional poverty [14,34], and deal with the impact of climate change. Food security in China and India is also of concern due to the large population bases of these countries.

### 3.6. Theme Analysis

**Figure 7:**
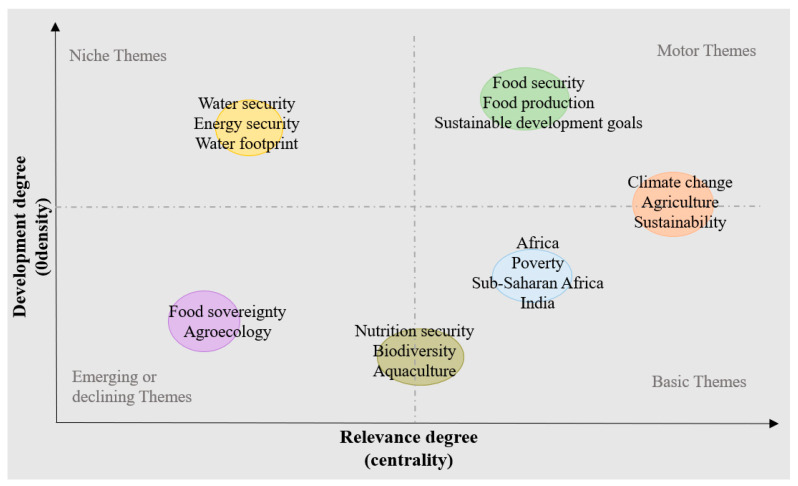
Distribution of themes in food security areas.

Theme mapping is a method that combines the advantages of traditional indexing and artificial intelligence, with keywords as the core of the data model to efficiently analyze its centrality and density, with the aim of exploring and reasoning about research topics. Theme maps can solve the problems caused by a large amount of disordered information. The horizontal and vertical axes indicate centrality and density, respectively, representing the workload and importance of the subject, respectively [35]. The theme map has four quadrants, and the current development stage of a theme can be analyzed by the location of the quadrant of the theme. Motor themes located in the first quadrant were both important and soundly trending, whereas niche themes in the second quadrant, although well developed, were of less importance to the field of food security. Emerging or declining themes in the third quadrant were marginal, with little workload and importance, whereas basic themes in the fourth quadrant were important for food security, with room for development (Figure 7).

Food production, climate change, and sustainability are currently the most popular themes, with high research density and centrality in the field of food security. The topics related to water security, energy security, water footprint, and the environment, whilst receiving a lot of attention, are not closely integrated with food security. The number and centrality of studies on food sovereignty, ecological agriculture, and childhood obesity were not high, indicating that these are emerging fields. In recent years, due to the impact of international conflicts and the COVID-19 pandemic, the issue of food sovereignty has received extensive attention [36]. Research on nutrition security, biodiversity, and aquaculture had a medium degree of centrality, but the degree of intensity was not high, indicating that although these topics were closely related to food security, the number of published papers was small, and the future research potential was high. Sustainable agricultural development and poverty eradication in Africa, Southeast Asia, and other regions are the basic research directions in the field of food security.

## 4. Discussion

### 4.1. Analysis of the Research Trend of Food Security

Research on food security concerns various areas, and the content of such research has been constantly enriched, with significant progress in recent years. Studies in this field are of great significance for judging the development trend of food security scientifically and rationally. The development and evolution of the concept of food security have obvious stage and historical characteristics. The understanding of the connotation of food security has been constantly enriched and improved, and the results have gradually changed from theoretical to applied results. At first, food security mainly emphasized the sufficiency of food production and supply. After that, studies in food security began to pay equal attention to the total amount of food and quality safety and to the balance of food supply and demand [5,6]. In recent years, scholars have focused on various food security issues, from simple quantitative security to nutrition security, life security, and ecological security.

The whole research and development process in the field of food security can be understood through the analysis of article references, high-frequency keywords, countries and institutions, and thematic maps. At present, the mainstream view is that the countermeasures and suggestions for food security should be discussed mainly based on the four pillars, availability, access, utilization, and stability. Climate change, food nutrition and security, and sustainable agricultural development are hot issues in the field of food security research. From the perspective of disciplines, the interdisciplinary trend of food security research is obvious, and a growing number of scholars in environmental science, demography, and geography are paying attention to food security. Some research fields focus on ensuring food security and putting forward corresponding countermeasures and suggestions. Scientists construct the evaluation index system of food security from the perspective of food production, distribution, consumption, and food reserves [37]. Among the many factors affecting food security, population is the most direct one, and the number of people directly affects the food supply pressure. The issue of food security in India and sub-Saharan Africa has received extensive attention from the international community. Historical reasons, natural resource constraints, climate disasters, agricultural production technology, and management level are all factors impacting food security in Africa. Taking them into account and exploring their interrelationships and influences can be the key to ensuring food security in Africa. It is worth noting that the research field has gradually expanded from the theoretical framework and norms to the technical level. More scholars in cross-cutting fields are considering how to promote food security from the technical level from the micro-perspective [38]. For example, they use the progress of biotechnology to improve varieties, promote efficient non-toxic pesticides, develop advanced agricultural technologies, improve water conservancy irrigation systems, establish agricultural information systems, and strengthen the application of information technology in agricultural management to achieve food security.

### 4.2. Sustainable Development Path of Food Security

The goal of food security research is to achieve food security in quantity, quality, and nutrition. Although the grain output still maintains a steady upward trend, and the nutritional level continues to improve, guaranteeing food security still faces enormous challenges, and the food security situation is still severe. Climate change poses many food security issues. Studies have shown that rising global temperatures and more extreme weather events will reduce the production of major food crops such as wheat, corn, and rice. To adapt to future climate change, adopting improved varieties, changing planting dates, and optimizing irrigation systems are important measures. Considering the productivity and sustainability of food and achieving the dual goals of food security and sustainable development are crucial topics. Agriculture accounts for 34% of greenhouse gas emissions, with the majority of them coming from land-use changes caused by agricultural activities and the rest from the food production supply chain [39]. Achieving the Sustainable Development Goals is important to resolve the contradictions between food production, greenhouse gas emissions, and resource depletion [40,41]. Man Li et al. proposed a comprehensive ecological and economic model to analyze crop phenology and nitrogen fertilizer absorption capacity, indicating that the yield can be improved by improving the efficiency of nitrogen fertilizer use [42].

Agro-ecosystems are heavily dependent on water resources, and an increase in food demand will inevitably lead to an increase in water demand [43]. Many scholars evaluated the importance of water resources security to food security [44,45]. Determining how to achieve food security under the premise of ensuring water resources security is an important research topic and involves technology, management, policy, and other aspects [36,46]. Looking at agricultural development from the perspective of sustainable development, it is necessary to explore ecological agriculture models while taking into account the protection of the ecological environment. For example, Lucantoni et al. analyzed the transition to eco-agriculture on a farm in Cuba [47]. Biodiversity conservation has been largely investigated by the international community, with an emphasis on balancing food security and biodiversity conservation. For example, JZA B et al. identified 10 hot-spots that would face food insecurity and biodiversity loss and called for attention to the status quo of conflict hot-spots in an effort to mitigate conflict [48,49]. Faced with the current situation that terrestrial grain productivity is constrained by future arable land potential and water resource depletion, Christopher et al. proposed that expanding the marine grain production capacity through timely fishery reforms would provide a guarantee for food security [50].

### 4.3. Future Research Direction

According to our analysis of the existing research results, we believe that the following issues in the field of food security will receive attention in the future:

(1) Improvement of the evaluation system for the food security level: The global food security measurement requires a complete indicator system. Demographic, climate, financial (agricultural subsidies, trade restrictions), and policy factors combined influence food security. In 2000, the Committee on World Food Security comprehensively considered consumption, health, and nutrition and formed seven monitoring indicators: (a) the incidence of undernourished population; (b) per capita dietary energy supply; (c) the proportion of grain and rhizome food calories in the per capita dietary energy supply; (d) life expectancy at birth; (e) mortality rate of children under 5 years; (f) proportion of underweight children under 5 years; (g) proportion of adults with a body mass index >18.5. Although the developed countries have sufficient access to food as a whole, local food insecurity is the main problem. For many developing countries, the root cause of food security is still food insufficiency caused by poverty. Numerous publications related to the indicator system have also been produced at national and regional levels, but the current evaluation indicator system is still not comprehensive enough and can only reflect part of the food security level. Food security at the micro-level has gradually become a hot topic, and the measures at the family and individual levels need to be further improved.

(2) Balancing sustainable development and food security: In the early stage, the degradation of natural resources, the decline in farmland production potential [51], and water shortage seriously threatened food security. Many studies focused on agricultural sustainability [52], and the determination of how to ensure food security under the premise of sustainable development is an important research direction at this stage.

(3) Research on improving agricultural production and management efficiency: There are still defects in grain production, sales, and management, along with an unreasonable allocation of resources and an untimely market supply. Improving the quality of agricultural products [53], adjusting the structure of agricultural production [54], and enhancing the popularity of large-scale mechanized agricultural production [55] are all methods to improve agricultural production efficiency. For example, PK Adom et al. showed that technical efficiency in Africa seriously restricted the potential of agricultural output and that technical support can largely contribute to food production in this region [56].

(4) Research on government food security policies and strategies: Currently, there are more severe food shortages in the world’s hunger hot-spots. In the context of intensive agricultural development, the rapid development of high-tech fields and the formation of technical barriers have widened the gap in the level of food security among countries. In the case of insufficient policy and institutional guarantees, along with the development of technology, the agricultural technology gap between developed and undeveloped countries will widen increasingly, making the poor population face a more serious food crisis. In the face of conflicts and disputes, natural disasters, and public health emergencies, the type of food security policies should also be the focus of sustainable food security research.

### 4.4. Comparative Analysis with Relevant Studies

The comparative analysis of relevant studies helps to determine differences, make up for deficiencies, and enrich research results. At present, research on food security involves factors such as concept, population, production, circulation, trade, policy, and the environment. The research perspective is multi-dimensional, and it can deeply discuss some specific issues. The literature is fruitful, and this research field is receiving increasing attention. However, the discussions on food security are scattered, ignoring the systematic characteristics of the research object, and it is difficult to form a unified logical main line connecting different research perspectives. We understood the development of food security research from a macro-perspective and noted the correlation between major research groups and countries as well as the distribution of research topics; we also analyzed the research results of major articles and authors. Our research attempts to clarify the cognitive context of academic circles on hot issues of food security help scholars understand the latest content of food security and can provide a reference for in-depth research on food security issues.

### 4.5. Limitations of the Study

This paper only used the core dataset in the Web of Science database as the data source. According to the retrieval conditions, the publications were from 1991 to 2022. Undeniably, there were some important research achievements before 1991. In addition, to exclude the interference of non-whole years with the research results, our research includes studies published before 31 December 2021. Therefore, we only analyzed the research results related to food security in the past 30 years. Our research is based on a single database, ignoring articles included in Scopus, CNKI, and other databases. In our following study, we will therefore explore how to incorporate other databases according to the current discussion, with the aim of expanding our research horizon.

## 5. Conclusions

In this study, we quantitatively analyzed the current state of research in the field of food security, based on the literature related to food security in the Web of Science database and combining the advantages of different bibliometric software packages. The results show that in the past 30 years, food security has experienced three stages of development, namely the initial stage, the development stage, and the high-yield and active stage; scholars’ understanding of the connotation of food security has been enriched and improved; and the research results have gradually changed from theory to application and are more practical and operable. The journals *Food Security*, *Sustainability*, and *Food Policy* published the largest numbers of articles and are dedicated to the discussion of food security issues, focusing on the publication of comprehensive views from interdisciplinary perspectives. Food security research covers 138 countries or regions, and the United States is at the vanguard in both quantity and quality. China is the developing country with the most significant contribution to food security research. Food security in sub-Saharan Africa, India, and other regions has received extensive attention. It is worth noting that most mature countries and institutions studying food security are located in Europe. Therefore, strengthening international cooperation in food security is the key to solving the world food problem. Climate change is the most frequently occurring keyword in the field of food security, followed by agriculture and management. The food security theme mapping shows that food production, climate change, and sustainable development had the highest research centrality degree and densities in the field of food security. Poverty in sub-Saharan Africa and India is an important theme for food security. At the same time, it is predicted that ecological agriculture, nutritional security, and food sovereignty will be research topics with good prospects.

To realize the world food security vision, the increasingly complex context of the global economy, including global climate change, production factors, technological progress, and international cooperation, needs to be considered. Maintaining and developing food security under the premise of sustainable development is the core of food security. Improving the food security index system, balancing the relationship between sustainable development and food security, improving the efficiency of agricultural production and management, and research on government food security policies and strategies will be the focus of the future.

## Figures and Tables

**Table 1:** Top 10 articles in the field of food security.

Title	DOI	Year	LCs	GCs
Food security: definition and measurement	10.1007/s12571-008-0002-y	2009	103	419
Conceptualizing food systems for global environmental change research	10.1016/j.gloenvcha.2007.09.002	2008	101	556
Towards better measurement of household food security: Harmonizing indicators and the role of household surveys	10.1016/j.gfs.2012.11.006	2013	59	157
Food security: a post-modern perspective	10.1016/0306-9192(95)00074-7	1996	56	255
The story of phosphorus: Global food security and food for thought	10.1016/j.gloenvcha.2008.10.009	2009	45	2722
A food systems approach to researching food security and its interactions with global environmental change	10.1007/s12571-011-0149-9	2011	45	266
Rethinking the measurement of food security: from first principles to best practice	10.1007/s12571-013-0253-0	2013	45	131
Lost food, wasted resources: Global food supply chain losses and their impacts on freshwater, cropland, and fertiliser use	10.1016/j.scitotenv.2012.08.092	2012	44	570
Climate change and food Systems	10.1146/annurev-environ-020411-130608	2012	43	904
Does adaptation to climate change provide food security? A micro-perspective from Ethiopia	10.1093/ajae/aar006	2011	42	493

Note: Local citations (LCs), number of citations in papers in this article’s database; global citations (GCs), number of citations in all papers.

**Table 2:** Top 7 journals published from 1991 to 2021 in the field of food security influence.

Journals	Articles	H-Index	TC	PY Start
*Food Security*	355	47	9701	2009
*Sustainability*	286	29	3685	2011
*Food Policy*	189	48	7453	1994
*Global Food Security*	155	39	3685	2008
*World Development*	105	36	4099	1997
*International Journal of Environmental Research and Public Health*	103	18	1179	2009
*Frontiers in Sustainable Food Systems*	85	10	410	2018

Note: H-index is a quantitative indicator of the number and level of academic output. The higher the value, the greater the impact of the journal in this field. TC is the total citations. PY Start is the year when the journal began to publish articles in the field of food security.

## Data Availability

Not applicable.
